# Misoprostol combined with artificial membrane stripping showed the best effect of induction among women with gestational diabetes mellitus: a retrospective cohort study

**DOI:** 10.1590/1806-9282.20251162

**Published:** 2025-12-15

**Authors:** Xuemei Li, Qimei Yang, Xia Zhang, Lidan He, Jianbo Wu

**Affiliations:** 1 Fujian Medical University, The First Affiliated Hospital, Department of Obstetrics and Gynecology – Fuzhou, China.; 2 Fujian Medical University, Binhai Campus of the First Affiliated Hospital, National Regional Medical Center, Department of Obstetrics and Gynecology – Fuzhou, China.; 3 The First People's Hospital of Yunnan, Department of Obstetrics – Kunming, China.

**Keywords:** Gestational diabetes mellitus, Misoprostol, Labor induction, Cervical ripening

## Abstract

**OBJECTIVE:**

The aim of this study was to explore the optimal method of induction of labor in women with gestational diabetes mellitus and its impact on delivery outcomes.

**METHODS:**

This retrospective cohort study was conducted among nulliparous women with gestational diabetes mellitus delivering at the First Affiliated Hospital of Fujian Medical University and the First People's Hospital of Yunnan Province from 2018 to 2023. Data were extracted from electronic medical records.

**RESULTS:**

A total of 600 patients who delivered met the inclusion criteria: the double-balloon group (Group B, n=198), the misoprostol vaginal insert group (Group M, n=200), and the Misoprostol combined with artificial membrane stripping group (Group MA, n=202). Group MA showed higher induction of labor success rates (93.5%) compared to Group M (86.0%) and Group B (81.5%, p=0.002). The time to labor onset was shortest in Group MA (32.5±6.5 h), followed by Group M (35.8±2.5 h) and Group B (45.8±4.5 h, p<0.001). Vaginal delivery time was also significantly shorter in Group MA (47.4±4.3 h) compared to Group M (49.1±7.4 h) and Group B (57.1±5.7 h, p<0.001). Chorioamnionitis rates were lower in Group MA (2.5%) and Group M (3.0%) compared to Group B (8.5%, p=0.007). No significant differences were observed between groups for cesarean rates, fetal distress, abnormal labor, or neonatal outcomes (p>0.05).

**CONCLUSION:**

The combination of misoprostol vaginal insert and artificial membrane stripping effectively enhances cervical ripening and improves vaginal delivery success in gestational diabetes mellitus patients, providing a promising strategy for labor induction.

## INTRODUCTION

Gestational diabetes mellitus (GDM) is a prevalent pregnancy complication, with an estimated global prevalence of 13.4%, rising to 17% in China^1^. Despite advances in its management through diet and exercise, GDM remains associated with severe pregnancy and delivery complications, including macrosomia, shoulder dystocia, and postpartum hemorrhage^2^. Effective IOL is crucial for reducing these risks in women eligible for vaginal delivery^3^.

The success of IOL heavily depends on cervical readiness, quantified by the Modified Bishop score. Pharmacological and mechanical methods are often employed for cervical ripening. Misoprostol, a prostaglandin E1 analogue, has shown promise in cervical ripening and labor induction due to its ability to mimic natural processes^4^, while AMS and double balloon offer mechanical adjuncts that promote endogenous prostaglandin release^5^.

Two previous studies on pharmacological IOL in women with GDM and normal pregnant women have shown that GDM slightly prolongs or does not alter the labor duration of misoprostol induction, and does not increase the risk of maternal-fetal complications, proving the efficacy and safety of misoprostol for induction in this population. However, there is still a lack of comparison of induction methods for pregnant women with GDM, and the debate on which is superior between mechanical and pharmacological methods remains.

This retrospective cohort study investigates the efficacy and safety of different methods for IOL in GDM pregnancies. By evaluating maternal and neonatal outcomes, we aim to determine the potential of this combination to enhance labor induction success, reduce complications, and support vaginal delivery in a high-risk population.

## METHODS

### Study subjects

**Figure 1:**
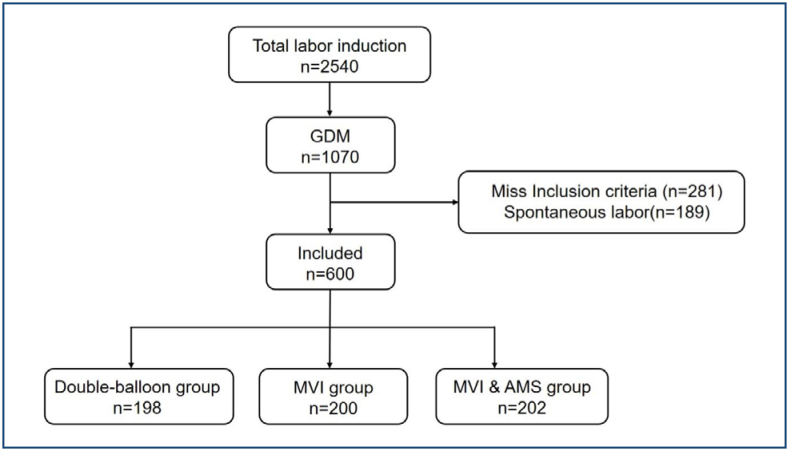
Trial profile of the study (AMS: artificial membrane stripping; GDM: gestational diabetes mellitus; MVI: misoprostol vaginal insert).

This retrospective cohort study involved 900 pregnant women with singleton pregnancies and poor cervical conditions (Bishop score<6) who were scheduled to be induced before the expected date of delivery (39–40 weeks of gestation) in the obstetrics departments of the First Hospital of Fujian Medical University and the First People's Hospital of Yunnan Province. Six hundred primigravid women who met the basic requirements, all of whom were GDM patients with full-term singleton cephalic pregnancies, were finally selected and categorized into the double balloon catheter group (198 cases in group B), the misoprostol group (200 cases in group M), and the misoprostol combined with manual stripping of the fetal membranes group (202 cases in group MA), respectively (Figure 1). The study followed the relevant requirements of the Declaration of Helsinki and was approved by the Ethics Committee of the First Affiliated Hospital of Fujian Medical University (MRCTA, ECFAH of FMU [2021]148).

Inclusion and exclusion criteria

Inclusion criteria:

1. Singleton pregnancy with cephalic presentation
2. Primigravid women (first-time mothers)
3. Gestational age ≥39 weeks
4. Diagnosed with GDM based on standard diagnostic criteria
5. Unruptured membranes
6. Cervical Bishop score<6 points before induction

Exclusion criteria:

1. Cephalopelvic disproportion (CPD)
2. Presence of severe maternal medical or surgical comorbidities or mental disorders that prevent cooperation
3. Pre-existing diabetes mellitus (type 1 or type 2 diabetes diagnosed before pregnancy)

A flow chart depicting the study inclusion and exclusion process is provided in Figure 1.

### General conditions

The three groups of pregnant women before IOL had a routine antenatal examination, body temperature and blood regular, leucorrhea examination without vaginitis, and fetal heart monitoring was reactive. In our study, the assignment of patients to each group was based on the clinical protocols and standard operating procedures (SOPs) of the two participating hospitals during the study period (2018–2023).

Group B (Double-balloon catheter): Patients were induced using the double-balloon catheter method according to the protocol followed at the First Affiliated Hospital of Fujian Medical University.

Group M (Misoprostol vaginal insert, MVI): Patients were induced using MVI as per the protocol of the First People's Hospital of Yunnan Province.

Group MA (Misoprostol combined with artificial membrane stripping, AMS): This combined method was adopted at the First People's Hospital of Yunnan Province as an optimized protocol for cervical ripening and labor induction in GDM patients with an unfavorable cervical vix.

The induction method selected was based on the institutional guidelines and clinical experience of the attending physicians at each hospital rather than randomization.

### Cervical balloon group

A double-lumen "Yixinda" CVB-18F disposable catheter was used in the cervical balloon group. After disinfection and cervical fixation, the catheter was inserted into the uterine cavity using toothed oval forceps, and the uterine balloon was filled with 20 mL of saline. The balloon was then withdrawn until it rested in the cervical canal, and another 20 mL was injected into the vaginal balloon. Following speculum removal, an additional 40 mL of saline was infused equally into both balloons, bringing the total to 80 mL. The catheter was secured to the inner thigh with gauze. Fetal heart rate monitoring followed placement. The balloon was removed immediately if membranes ruptured or contractions became excessively strong or frequent. If labor did not commence within 12 h, low-dose oxytocin was administered to induce contractions.

### Misoprostol vaginal insert group

The misoprostol group was manufactured by Wuhan Jiulong Renfu Pharmaceutical. Each misoprostol tablet is 0.2 mg, divided into 25 μg. After disinfecting the vulva, 25 μg of misoprostol is inserted into the posterior fornix of the vagina, and the patient should lie in bed for 30 min before getting out of bed to move. The maximum dose of the drug should be 50 μg within 24 h, and the drug should be discontinued if labor is imminent or if the membranes are ruptured. The drug can be used for 3 consecutive days, and if labor is not imminent on the third day, a small dose of serotonin should be given to induce labor.

### Misoprostol combined with the artificial membrane stripping group

After disinfecting the vulva and vagina with benzalkonium chloride, the index or middle finger is inserted into the inner mouth of the cervical canal, the fetal membranes are gently separated clockwise by pressing against the lower part of the uterus and rotating it 360°, at a depth of about 3∼5 cm, the fetal membranes are separated, and the cervical canal is dilated; if spongy tissue is touched or there is heavy bleeding, the procedure should be stopped immediately. After manual separation of the membranes, misoprostol 25 μg is placed in the posterior vaginal dome, and the procedure is the same as in the misoprostol group.

### Induction of labor after cervical ripening

Mechanical or pharmacological cervical ripening is no longer performed if the cervical Bishop score is ≥7 points. Add 2.5 IU of oxytocin to 500 mL of solvent and administer via intravenous infusion, or perform artificial rupture of membranes until labor is onset.

### Determination of failed induction of labor

From the time of premature or artificial membrane rupture, regular uterine contractions induced by oxytocin last for more than 18 h without the parturient entering the active phase, or a cesarean section is performed before entering the active phase.

### Observation indices

(1) Maternal labor outcomes: time from induction to labor onset, time to delivery, number of deliveries within 24 h, number of cases of cesarean section, rate of postpartum hemorrhage, and number of cases of chorioamnionitis. (2) Diagnostic criteria for chorioamnionitis infection: maternal temperature ≥37.8°C; accompanied by two or more of the following signs: maternal peripheral blood leukocyte count>15×10^9^/L; maternal heart rate>100 beats per min; uterine pressure pain; vaginal secretion odor; fetal heart rate>160 beats per min; and placental pathology showing that the diagnosis of chorioamnionitis or placental inflammation can be confirmed^6^. (3) Compare fetal and neonatal conditions during and after delivery.

### Statistical analyses

Statistical analyses were performed using GraphPad Prism 9.0 software (La Jolla, CA, USA), and normally distributed measures were presented in ±s. The independent samples t-test was used for comparisons between two groups, and one-way ANOVA was used for comparisons between three or more groups. Count data were presented as frequencies, and the χ^2^ test or Fisher's exact probability method was chosen as appropriate. p-values for all analyses, with p<0.05, were considered statistically significant differences.

## RESULTS

### Comparison of basic information

The three groups’ age, gestational week, Bishop score, and Oral Glucose Tolerance Test (OGTT) were confirmed to be normally distributed. When comparing the general information of the pregnant women in the three groups, the difference was not statistically significant (p>0.05) (Table 1).

**Table 1:** Baseline demographics and pregnancy characteristics.

Characteristics		Group B (n=198)	Group M (n=200)	Group MA (n=202)	p
Maternal age (years)		28.5±3.1	28.2±3.9	28.9±4.0	0.160
Gestational age (week)		39.3±1.1	40.3±0.2	40.2±0.4	0.997
Prepregnancy BMI (kg/m^2^)					0.152
	≥25	30	45	40	
	<25	168	155	162	
Bishop Score (point)		3.5±0.71	3.7±0.5	3.1±0.9	1.000
OGTT (mmol/L)					
	0 h	4.36±1.13	3.9±1.1	4.7±1.2	1.000
	1 h	9.36±1.13	10.4±2.1	9.1±1.9	1.000
	2 h	7.36±1.13	8.5±1.8	8.2±2.1	1.000

Group B: double-balloon group; Group M: misoprostol vaginal insert group; Group MA: Misoprostol combined with artificial membrane stripping group; BMI: body mass index; OGTT: Oral Glucose Tolerance Test; p<0.05 was considered significant.

### Comparison of efficacy and safety among different labor induction methods

As shown in Table 2, the MA group demonstrated superior efficacy compared to groups M and B. The induction success rate in the MA group reached 93.5%, significantly higher than in groups M (86.0%) and B (81.5%) (p=0.002). The MA group also exhibited the shortest mean times to labor onset (32.5±6.5 h) and to delivery (47.4±4.3 h), both significantly lower than in the other two groups (p<0.001). Furthermore, misoprostol administration exceeding four doses occurred in only 20.5% of MA cases, markedly lower than the 36.0% observed in the M group (p=0.005), indicating improved efficiency with reduced drug exposure. Regarding safety, no significant differences were noted among the three groups in the incidence of postpartum hemorrhage or major complications such as uterine rupture, placental abruption, or umbilical cord prolapse. Importantly, chorioamnionitis was significantly less frequent in groups M and MA than in group B (p=0.007). The rates of cesarean section due to intrauterine distress, abnormal labor, or failed induction were comparable across groups. These findings suggest that misoprostol combined with AMS is the most effective and safest method among the evaluated induction strategies.

**Table 2:** Comparison of labor outcomes and complications.

Characteristics		Group B (n=198)	Group M (n=200)	Group MA (n=202)	p
Success rate of IOL (n, %)		163 (81.5%)	172 (86.0%)	187 (93.5%)	0.002
Time to labor onset (h)^a^		45.8±4.5	35.8±2.5	32.5±6.5*	<0.001
Time to delivery (h)		57.1±5.7	49.1±7.4	47.4±4.3*	<0.001
Vaginal delivery within 24 h (n, %)^b^		6 (3.0%)	9 (4.5%)	11 (5.5%)	0.466
Postpartum hemorrhage (n, %)		20 (10.0%)	18 (9.0%)	16 (8.0%)	0.783
Chorioamnionitis (n, %)		17 (8.5%)	5 (2.5%)	6 (3.0%)	0.007
Main indications for cesarean section					
	Intrauterine distress (n, %)	36 (18.0%)	40 (20.0%)	45 (22.5%)	0.532
	Abnormal Labor (n, %)	23 (11.5%)	25 (12.5%)	20 (10.0%)	0.730
	Failed IOL (n, %)	20 (10.0%)	19 (9.5%)	21 (10.5%)	0.946
	mVisual score of pain	8	5	6	<0.001
Neonatal outcomes and complications					
	Amniotic fluid III (n, %)	45 (22.5%)	51 (25.5%)	57 (28.5%)	0.378
	Neonatal asphyxiation (n, %)	7 (3.5%)	8 (4.0%)	8 (4.0%)	0.956
	Hypoglycemia (n, %)	20 (10.0%)	31 (15.5%)	29 (14.5%)	0.226
	Weight (g)	3,210±415	3,180±430	3,228±412	0.512

Group B: double-balloon group; Group M: misoprostol vaginal insert group; Group MA: Misoprostol combined with artificial membrane stripping group; IOL: induction of labor.

a Excludes Caesarean sections and failed inductions of labor.

b Excludes Caesarean sections.

m Quantitative data are presented as mean±standard deviation, while median and range are provided for ordinally scaled parameters. "-" indicates no data available.

* denotes a statistically significant difference with p<0.05 when comparing Group M and Group MA.

The statistical significance level was set at p<0.05. If p>0.001, it is reported to three decimal places. If p≤0.001, it is reported as p<0.001.

### Comparison of neonatal outcome

Considering the incidence of amniotic fluid III, neonatal hypoglycemia, asphyxia, and low birth weight, there was no significant difference among the three induction methods (p>0.05) (Table 2).

## DISCUSSION

This study demonstrated that the combination of misoprostol and AMS significantly enhances cervical ripening in pregnant women with GDM, achieving a success rate of 93.5%. This was notably higher than the rates observed with misoprostol alone (86.0%) or the double-balloon catheter (81.5%). Furthermore, the combination significantly shortened the induction-to-delivery interval and reduced the incidence of complications such as chorioamnionitis (2.5% in the MA group vs. 8.5% in the double-balloon group). These findings highlight the efficacy and safety of this combined approach, offering a promising alternative for optimizing labor induction strategies in GDM patients.

The results of this study align with and extend previous findings. Duffy highlighted prolonged induction times and higher rates of neonatal jaundice in GDM patients undergoing induction with dinoprostone vaginal inserts, underscoring the need for optimal induction methods for this population^7^. Previous evidence emphasized the diverse applications of misoprostol in induction. However, it noted the lack of consensus on the optimal method^4,8,9^. This study innovatively combined AMS with misoprostol, showing superior efficacy to single-agent or mechanical methods.

Compared to mechanical methods like the Cook cervical ripening balloon (CCRB), AMS offers simplicity and lower costs. Studies by Wang and Duro Gomez supported the effectiveness of mechanical methods but also noted potential complications such as cervical trauma and increased pain^10,11^. This study demonstrated that when combined with misoprostol, AMS reduces the frequency of intervention and mitigates these risks. Findings by Heilman^12^, which confirmed AMS's safety as an induction method, are consistent with our results, further validating the clinical utility of this technique.

The synergistic effect of misoprostol and AMS is a key factor in the improved outcomes observed in this study. Misoprostol mimics endogenous prostaglandins, promoting cervical softening and uterine contractions^13^, while AMS mechanically stimulates additional prostaglandin release. This "dual-action" approach accelerates the natural ripening process, reducing the need for repeated misoprostol doses. A previous study has highlighted the benefits of combining pharmacological and mechanical methods for enhancing Bishop scores and induction efficiency^14^. It is worth noting that our misoprostol dosing regimen was more conservative than that recommended by the FIGO 2023 guidelines (25–50 23 guideline)^15^. This approach was adopted to ensure maternal safety, particularly in women with GDM who may be more prone to uterine hyperstimulation. The drug was discontinued immediately if labor was imminent or if membranes ruptured. Future studies should explore whether a more aggressive dosing strategy, in line with international guidelines, can be safely applied in this high-risk population.

This study involved a well-defined cohort of pregnant women with GDM, applying strict inclusion and exclusion criteria to ensure comparability across groups. The use of a standardized induction protocol enhanced the reliability of outcome assessments. In contrast to many prior studies, this investigation comprehensively evaluated both efficacy and safety, including key complications such as chorioamnionitis, thereby offering robust support for the proposed induction strategy. Nonetheless, several limitations must be acknowledged. Although our study population was selected based on strict inclusion criteria, including singleton cephalic presentation and Bishop score<6, and baseline characteristics were comparable across groups, we did not specifically control for or stratify by these variables in the statistical analysis. Therefore, residual confounding cannot be entirely ruled out. Future prospective studies should consider incorporating these factors into the analysis better to isolate the actual effect of the induction methods. Additionally, subgroup analyses based on BMI categories or GDM severity could provide more nuanced insights into how these variables interact with the efficacy and safety of different induction strategies. The retrospective design may introduce selection bias, as the choice of induction method was clinician-determined rather than randomized, potentially overestimating the efficacy of the combined misoprostol and AMS approach. Cervical readiness was assessed using the Bishop score, which is subjective and susceptible to interobserver variability. Inclusion of patients from two hospitals may introduce variability in clinical practice, limiting generalizability. The study's focus on GDM patients in a specific region, the absence of long-term neonatal outcome data, and the small sample size for specific subgroups further constrain broad applicability. Future prospective randomized trials with larger, more diverse populations are needed to confirm these findings.

The findings emphasize the practicality of combining pharmacological and mechanical methods for labor induction in GDM patients. Misoprostol combined with AMS represents a time-efficient, effective strategy for improving cervical ripening and reducing complications. These results provide valuable guidance for obstetricians in optimizing induction protocols, particularly for high-risk populations such as women with GDM. Future studies should investigate the applicability of these findings across a broader population and different gestational ages. Multicenter randomized controlled trials (RCTs) are needed to determine the optimal dosage, timing, and conditions for AMS use in various clinical scenarios. Additionally, research on patient satisfaction and long-term neonatal outcomes will provide further insights into the value of this combined approach.

## CONCLUSION

This study demonstrates that combining misoprostol with AMS significantly improves cervical ripening success rates, shortens induction-to-delivery time, and minimizes complications in GDM patients. As a safe and effective induction method, this approach offers significant advantages in managing high-risk pregnancies and provides strong evidence for optimizing induction strategies in clinical practice. However, retrospective cohort studies remain a valuable and commonly used approach in clinical research, especially when prospective RCTs are not feasible due to ethical, logistical, or practical constraints. In this study, we aimed to evaluate real-world clinical practices and outcomes in labor induction among women with GDM. Retrospective data collection allowed us to analyze a large and well-characterized cohort, providing meaningful insights into the comparative effectiveness and safety of different labor induction methods in this high-risk population. However, the findings may require further prospective validation.

## Data Availability

The datasets generated and/or analyzed during the current study are available from the corresponding author upon reasonable request.
